# Action of Salicylates in Acute Rheumatism

**Published:** 1906-12-01

**Authors:** 


### ACTION OF SALICYLATES IN ACUTE RHEUMATISM.

In a clinical lecture on the action of salicylates in acute rheumatism Dr. Ralph Stockman, Professor of Materia Medica in the University of Glasgow, claims for these drugs a specific effect upon the course of the disease, and compares their action to that of mercury in syphilis, quinine in malaria, and the serums in other diseases. He explains their rapid action on the pain and swelling of the joints by the fact that they are excreted into the joint cavity from the blood in comparatively large quantities, as can be shown by their detection in the fluid withdrawn by means of a hypodermic syringe. On the other hand he thinks their failure to exert the same influence on affections of the cardiac valves or fibrous tissues is due to the inability of the drugs to reach these parts in the same way. The experimental work of Walker and Ryffel at Guy's Hospital tends to confirm this view of the specific power of salicylates in rheumatic fever. They showed that the rheumatic bacillus decomposes lactic acid into acetic and formic acids and that this action is inhibited by salicylic acid. This inhibition is probably due to the formation of an "ester" compound between the salicylic and lactic acids. Formic acid is present in normal urine in minute quantities, but is greatly increased in quantity in rheumatic fever. When, however, the patient is flooded with salicylic acid under the ordinary treatment, the formic acid in the urine is rapidly diminished to the normal amount. Walker and Ryffel found that salicylic acid has no direct bactericidal action on the rheumatic bacillus. On the other hand clinical experience does not altogether support the claim of specificity for salicylates, and some of our highest authorities have formed a quite contrary opinion from their experience of the course of the disease under treatment with these drugs. The rapid subsidence of all joint trouble on the administration of salicylates is, of course, admitted on all sides, but the actual disease still lurks in the body of the patient, as shown first by the frequent relapses which occur as soon as the treatment in regard to diet and rest is in any way relaxed, and again by the liability to the later development of valvular disease of the heart. If, however, salicylates do not destroy the rheumatic bacillus, or if, as Dr. Stockman suggests, they are unable to reach certain regions of the body when the disease has already gained a foothold, one can easily understand how these subsequent relapses and sequelæ may arise. There is, therefore, a considerable danger in laying too great a stress upon the specificity of the salicylates, for it may be readily assumed in consequence that with the subsidence of all symptoms of joint trouble the disease has been cured, and the patient is accordingly allowed that liberty of action which it is so essential to prohibit in order to guard against subsequent valvular affections. It is not unlikely that in this way the opinion has been formed in some quarters that the use of salicylates in rheumatic fever actually exerts a deleterious effect upon the cardiac tissues and tends to increase their liability to rheumatic lesions. It must be the experience of all physicians that great firmness and tact are frequently required to induce a patient to remain at rest, when all pain and discomfort have been completely removed by salicylate administration.

